# Gene Regulation System of Vasopressin and Corticotoropin-Releasing Hormone

**DOI:** 10.4137/grsb.s424

**Published:** 2008-03-03

**Authors:** Masanori Yoshida

**Affiliations:** Department of Endocrinology, Nagoya Ekisaikai Hospital, 454-8502, Japan. Department of Endocrinology and Diabetes, Nagoya University Graduate School of Medicine, Nagoya 466-8550

## Abstract

The neurohypophyseal hormones, arginine vasopressin and corticotropin-releasing hormone (CRH), play a crucial role in the physiological and behavioral response to various kinds of stresses. Both neuropeptides activate the hypophysial-pituitary-adrenal (HPA) axis, which is a central mediator of the stress response in the body. Conversely, they receive the negative regulation by glucocorticoid, which is an end product of the HPA axis. Vasopressin and CRH are closely linked to immune response; they also interact with pro-inflammatory cytokines. Moreover, as for vasopressin, it has another important role, which is the regulation of water balance through its potent antidiuretic effect. Hence, it is conceivable that vasopressin and CRH mediate the homeostatic responses for survival and protect organisms from the external world.

A tight and elaborate regulation system of the vasopressin and CRH gene is required for the rapid and flexible response to the alteration of the surrounding environments. Several important regulatory elements have been identified in the proximal promoter region in the vasopressin and CRH gene. Many transcription factors and intracellular signaling cascades are involved in the complicated gene regulation system. This review focuses on the current status of the basic research of vasopressin and CRH. In addition to the numerous known facts about their divergent physiological roles, the recent topics of promoter analyses will be discussed.

## Introduction

In a severe environment, all organisms are constantly exposed to a wide variety of stresses, which sometimes even threaten their lives. Therefore, the acquisition of the ability to adapt to the environmental changes probably gives them a great advantage for survival. The way in which adaptation occurs can be divided into some different types, such as physiological or behavioral response. The former includes an autonomic response such as fluid balance, vasoregulation, and energy homeostasis, most of which are biologically fundamental responses for the maintenance of the internal milieu. The immune response as a defense system may also be added here. The latter, a behavioral response to the external world, often becomes a basic strategy to win the struggle for existence. It includes fight and flight, eating and drinking, memory and learning, or emotional reactions such as fear and anxiety. For this purpose, whatever the response, the construction of an acute and accurate “cell-to-cell” or “organ-to-organ” communication system is required. Indeed, the highly-developed signal transduction system in the body is considerably orchestrated by the endocrine system.

The concept of stress was first advocated by Hans Selye (Selye, 1936; Selye, 1946). He noticed the importance of the adrenal cortex in the stress response from the observation that it was significantly enlarged under the stressful condition. Subsequently, numerous works have provided us with extended knowledge about the profound relationship between stress and the endocrine system (Besedovsky and del Rey, 1996; Turnbull and Rivier, 1999). In vertebrates, after stress is sensed by the peripheral organs, much information is gathered to the hypothalamus in the brain, and then many neuropeptides and neurotransmitters begin to make appropriate and flexible responses in accordance with environmental changes. In this neuroendocrine response to the external world, in particular, hypothalamic vasopressin and corticotropin-releasing hormone (CRH) play a central role. Both neuropeptides are essential to keeping the life homeostasis.

Vasopressin is known as an antidiuretic hormone regulating water balance. The principal role of vasopressin is the maintenance of the plasma osmolality (285mOsm/H_2_O) by controlling water reabsorption in the kidney (Robertson, 1976; Schrier et al. 1979; Burbach et al. 2001). However, vasopressin possesses more divergent physiological actions, such as vasoconstriction and interaction with anti-inflammatory mediators. On the other hand, CRH stimulates the rapid release of adrenocorticotropic hormone (ACTH) from the anterior pituitary (Rivier and Plotsky, 1986; Orth, 1992). Then, ACTH stimulates the glucocorticoid synthesis and secretion from the adrenal cortex. Glucocorticoid exerts a pleiotropic effect on energy metabolism, vasoregulation, and the immune system (Munck et al. 1984; Sapolsky et al. 2000; Buckingham, 2006). Therefore, CRH is thought to act as an upstream regulator of this important cascade, the hypophysial-pituitary-adrenal (HPA) axis (Bale and Vale, 2004; Majzoub, 2006). Of particular interest is the fact that vasopressin enhances this stimulatory effect of CRH on ACTH secretion (DeBold et al. 1984; Antoni, 1993). In addition to the HPA axis activation, CRH directly regulates the feeding behavior and the emotional reaction in the brain: CRH induces the anorexigenic and anxiogenic reaction. Food intake is rapidly reduced (within 30min) after the central administration of CRH in rodents (Arase et al. 1988). This acute anorexigenic effect of a single injection does not persist for a long duration; however, the continuous infusion of CRH promotes body weight loss. CRH is also distributed in the extra-hypothalamic region including the amygdala and the bed nucleus of the stria terminalis (BNST), both of which are closely associated with fear and anxiety (Walker et al. 2003), suggesting that CRH mediates the behavior in unfamiliar surroundings (Merali et al. 2004; Bale and Vale, 2004). On the other hand, the dys-regulation of CRH in these regions probably causes depression or mood disorders (Stenzel-Poore et al. 1994; Risbrough and Stein, 2006).

Thus, vasopressin and CRH are involved in the adaptation to the internal or external stress condition. Then, how is the gene expression of vasopressin and CRH controlled, in response to this broad range of stresses? There are common transcription factors and intracellular signaling pathways for the strict gene regulation of both neuropeptides. This paper focuses on the current status of the basic research regarding the physiological role and the gene regulation system of these neuropeptides.

## Structure

Vasopressin is a representative posterior pituitary hormone which was first discovered by du Vigneaud and his co-workers in the 1950s (Popenoe and du Vigneaud, 1954). The human vasopressin gene is composed of three exons and two introns, and is encoded in chromosome 20 (Ivell and Richter, 1986). The structures of vasopressin and oxytocin, another posterior pituitary hormone, resemble each other. Interestingly, the vasopressin-coding region is at a distance of 12 kbp from that of the oxytocin gene in the opposite direction in the same chromosome, strongly suggesting that oxytocin is a duplicated gene of vasopressin.

Like many other neuropeptides, the bioactive form of vasopressin is generated from a large precursor, preprovasopressin (Burbach et al. 2001). After the removal of the signal peptide, provasopressin is further processed into the three end products, neurophysin II, glycoprotein, and mature vasopressin by intracellular processing. The bioactive vasopressin has a cyclic structure due to an intramolecular disulfide bond between two cysteines. Neurophysin II is a unique cystein-rich polypeptide, known as an important carrier protein which supports the axonal transport of vasopressin in the vasopressinergic neurons (Gainer et al. 1977). On the other hand, the role of glycoprotein still remains unclear.

CRH is a 41-amino-acid neuropeptide which was first isolated from sheep hypothalamus (Vale et al. 1981). The human CRH gene is composed of two exons and one intron, encoded in chromosome 8. The CRH coding region starts in exon2 and first of all, preproCRH is synthesized. As with vasopressin, after the removal of the signal peptide, the mature CRH peptide is generated by further processing of proCRH and N-terminal amidation. To date, CRH is known to belong to the CRH peptide family, along with urocortin1 (Ucn1) (Vaughan et al. 1995), Ucn2 (Reyes et al. 2001), and Ucn3 (Lewis et al. 2001). Ucns were originally identified as other ligands of CRH receptors. Mouse Ucn1 is approximately 45% identical to CRH (Vaughan et al. 1995), whereas mouse Ucn2 shares 34% and 42% amino acid homology with rat CRH and rat Ucn1, respectively (Reyes et al. 2001). On the other hand, Ucn3 is more distantly related to Ucn1 and CRH: mouse Ucn3 is 18% identical to mouse Ucn1, 40% to mouse Ucn2, and 26% to rat CRH (Lewis et al. 2001).

## Distribution and Receptors

The tissue distribution of the vasopressin gene is almost limited to the central nervous system, with a few exceptions. Vasopressin is mainly located in the magnocellular neurons of the supraoptic nucleus (SON), magnocellular and parvocellular parts of the paraventricular nucleus (PVN), and suprachiasmatic nucleus (SCN) of the hypothalamus. Vasopressin-positive neurons are also recognized in the forebrain area, such as BNST and the medial amygdaloid nucleus (van Leeuwen and Caffe, 1983). Each discrete region plays a different physiological role through the three different vasopressin receptors, such as V1a (Morel et al. 1992), V1b (Sugimoto et al. 1994), and V2 (Lolait et al. 1992) receptor. In the peripheral tissues, the vasopressin gene expression is detected in the thymus, testis and vasculature.

Compared with vasopressin, CRH is more extensively distributed in many brain areas, the spinal cord and peripheral tissues (Cummings et al. 1983; Keegan et al. 1994). In the hypothalamus, CRH-positive neurons are mainly recognized in the parvocellular subdivision of PVN, some of which are co-localized with vasopressin. In contrast, there are few CRH-positive fibers in the magnocellular subdivision of PVN and SON, where vasopressin is strongly expressed (Itoi et al. 2004). The CRH gene expression is also observed in the cerebral cortex, limbic system (amygdala, hyppocampus, and BNST), thalamus, cerebellum, brainstem, and dorsal root of the spinal cord. In the periphery, CRH is present in the adrenal medulla (Hashimoto et al. 1984), gastrointestinal tract (Nieuwenhuijzen Kruseman et al. 1982), skin (Slominski et al. 1998), and placenta (Petraglia et al. 1987); most of these organs are deeply associated with stress or immune responses.

The physiological effect of CRH and related peptides such as Ucns is exerted via CRH receptors, which belong to the seven-transmembrane, G-protein coupled receptors (GPCR). To date, two subtypes of CRH receptor, CRHR1 (Chen et al. 1993) and CRHR2 (Lovenberg et al. 1995) have been identified. They have approximately 70% similarity at the amino acid level. In addition, eight splice variants of CRHR1 (a-h isoforms) (Pisarchik and Slominski, 2001) and three splice variants of CRHR2 (α, β and γ isoforms) (Catalano et al. 2003) have already been characterized. CRHR1 is distributed throughout the brain, in areas such as the cerebral cortex, cerebellum, olfactory bulb, hippocampus, amygdala, and more importantly, anterior pituitary corticotroph. CRHR2α expression is restricted to the brain, in areas such as the hypothalamus, lateral septum, and hippocampus, whereas CRH-R2β is widely expressed in the peripheral tissues, including the heart, lung, gastrointestinal tract, and skeletal muscle. In contrast, CRHR2γ is only detected in the human brain. CRH mainly binds CRHR1, but not CRHR2. On the other hand, all Ucns bind CRHR2 with high affinity. Ucn1 have equal affinities for CRHR1 and CRHR2; however, neither Ucn2 nor Ucn3 binds CRHR1. The above observation suggests that each of the CRH and Ucns exerts different physiological functions in the different target tissues via these receptors.

In the systemic circulation, CRH and Ucn1 are known to bind CRH-binding protein (CRH-BP), resulting in the inactivation of CRH and Ucn1 (Potter et al. 1991). In contrast, Ucn2 and Ucn3 cannot form the dimer complex with CRH-BP. The mRNA of CRH-BP is detected in the cerebral cortex, limbic systems including amygdala, brainstem, and pituitary corticotroph (Potter et al. 1992). In addition, CRH-BP is also expressed in the placenta (Petraglia et al. 1993). CRH-BP blocks the inappropriate activation of the HPA axis in pregnant women, because circulating CRH derived from placenta progressively increases during pregnancy (Sasaki et al. 1984). In CRH-BP transgenic animals, the plasma concentration of ACTH and corticosterone is not reduced, despite the abundant expression of CRH-BP in the pituitary (Burrows et al. 1998). CRH and vasopressin mRNA in PVN is elevated in these mice, suggesting that they compensate for the deteriorated function of CRH in corticotroph and resultantly maintain the HPA axis.

## Physiological Roles

Potent antidiuresis is the most important physiological role of vasopressin. Vasopressin achieves the prevention of body fluid loss by regulating the reabsorption of water in the kidney. Therefore, vasopressin deficiency causes central diabetes insipidus (CDI) with marked polyuria at an amount of more than 10 liters per day. In the magnocellular neurons of SON and PVN, the vasopressin synthesis is stringently mediated by the plasma osmolality (Robertson et al. 1976). The magnocellular neurons perceive the osmotic stimuli through the excitatory glutaminergic and inhibitory GAB-Aergic neurons projected from the osmoreceptors. The osmoreceptor is a unique osmosensitive organ, located in the circumventricular region which includes the subfornical organ (SFO), organum vasculosum of the lamina terminalis (OVLT), and median preoptic nuclei (MnPO) (Andersson, 1977; Bourque and Oliet, 1997; Burbach et al. 2001; McKinley et al. 2004). Vasopressin is packaged within the secretory granules and transported into the nerve terminals of the magnocellular neurons. Vasopressin is first gathered in the posterior pituitary and then rapidly released into the general circulation in response to hyperosmotic change. In contrast, it is also presumed that the magnocellular neurons are themselves osmosensitive, because the osmotic change electrically evokes these vasopressinergic neurons (Mason, 1980; Oliet and Bourque, 1993; Zhang and Bourque, 2003). Unfortunately, the precise mechanism of the direct osmosensitivity of the magnocellular neurons has been unexplored, partly because isolated pure vasopressinergic neurons are not readily available.

The antidiuretic effect of vasopressin is achieved through the GPCR member, V2 receptor (V2R), which is expressed at the basolateral side of the tubules and the collecting ducts of the kidney (Lolait et al. 1992; Knepper, 1997; Robben et al. 2006). A rapid accumulation of the intracellular cAMP and the subsequent activation of protein kinase A (PKA) is observed, after vasopressin in the circulation binds V2R. Afterwards, a water channel, aquaporine2 (AQP2), which is present in the intracellular vesicles, is rapidly phosphorylated at the portion of Ser256 through the cAMP/PKA-dependent pathway (Fushimi et al. 1993; Kuwahara et al. 1995). The activated AQP2 migrates to the apical membrane of the principle cells of the collecting ducts. A large amount of water is reabsorbed through AQP2 and returned into the circulation through AQP3 and AQP4, present in the opposite basolateral site of these cells. Finally, AQP2 is dephosphorylated and internalized to the original stores in the cytoplasm. This sophisticated recycling system of AQP2 regulates the water balance in the body.

Vasopressin also maintains the cardiovascular system in response to hemodynamic stress (Johnston, 1985; Arnolda et al. 1986). Hypotension or hypovolemia initiates the secretion of vasopressin from the posterior pituitary through the baroreceptor in the aortic arch and chemoreceptor in the carotid body. In fact, the name “vasopressin” comes from the vasopressor action. Vasopressin increases the arterial blood pressure through the V1a receptor (V1aR), which exists in the vessel wall (Morel et al. 1992; Koshimizu et al. 2006). Clinically, vasopressin is recently being used as a potent hypertensive drug, as are cathecholamines, for the rescue of patients in shock in the emergency room (Wenzel and Lindner, 2006). In addition, the V1aR gene expression is also observed in many brain areas, where vasopressin promotes social behavior, learning and memory (Everts and Koolhaas, 1999; Bielsky et al. 2004). Vasopressin causes aggression or paternal behavior through the brain V1aR, in contrast to oxytocin, which causes maternal behavior (Bielsky and Young, 2004). Otherwise, several vasopressin derivatives which enhance the memory retention have been developed (Hirate et al. 1997).

Interestingly, there are some common physiological actions in vasopressin and CRH, even though their molecular structure do not resemble each other. Vasopressin and CRH are deeply associated with the neuroimmunomodulation system, because they activate the HPA axis playing a principal role in stress and immune response (Aguilera, 1994; Turnbull and Rivier, 1999). CRH and vasopressin act as ACTH secretagogues (DeBold et al. 1984; Rivier et al. 1984; Antoni, 1993). In PVN, the parvocellular neurons expressing CRH and vasopressin extend to the corticotroph cells in the anterior pituitary through the median eminence (Holmes et al. 1986). CRH promotes the rapid release of ACTH from the corticotroph via the CRHR1. Then, ACTH stimulates the glucocorticoid synthesis and secretion from the adrenal cortex. In addition, CRH is also capable of stimulating the adrenal gland directly and inducing glucocorticoid production. Almost all CRH-positive neurons do not express vasopressin in the basal condition; however, a number of these neurons start to express vasopressin after receiving the stress, resulting in the increase of the ACTH secretion via the corticotroph V1b receptor (V1bR), as is the case with CRH. Compared with CRH, vasopressin itself has a weak ability of up-regulating the ACTH secretion; however, it markedly potentiates the effect of CRH (Gilles et al. 1982; DeBold et al. 1984; Volpi et al. 2004). Altogether, CRH and vasopressin are characterized as upstream regulators of the HPA axis.

The synthesis and secretion of hypothalamic CRH and vasopressin, in turn, receive negative feedbackbytheexcessiveproductionofglucocorticoid. There is abundant evidence regarding the suppression of CRH and vasopressin gene expression by glucocorticoid. Adrenalectomy induces the CRH mRNA expression in the PVN; however, the CRH gene expression was decreased by glucocorticoid replacement (Jingami et al. 1985; Bayer et al. 1988). On the other hand, CRH heteronuclear (hn) RNA and mRNA expression are significantly decreased in adrenalectomized rats, after stress stimulation (Tanimura and Watts, 1998). The latter interesting observation suggests that a physiological concentration of glucocorticoid is important for the stress-induced CRH expression. It is strongly assumed that the glucocorticoid receptor (GR), which is expressed in PVN and pituitary corticotroph, is involved in this negative feedback system. In SON, the GR expression is undetectable under normal conditions, but it becomes recognizable under chronically hypoosmotic stimulation (Berghorn et al. 1995). Interestingly, the CRH production is up-regulated by glucocorticoid in other tissues such as the amygdala (Makino et al. 1994a), BNST (Makino et al. 1994b) and placenta (Robinson et al. 1988). Aside from this, other investigators report that the CRH gene expression in the amygdala is unchangeable by glucocorticoid (Kasckow et al. 1997). In any case, the precise mechanisms of these tissue-specific regulations of CRH gene expression by glucocorticoid have not been fully elucidated.

After adrenalectomy, vasopressin immunoreactivity is markedly elevated in the CRH-positive, parvocellular neurons in PVN (Sawchenko et al. 1984; Kiss et al. 1984). A significant increase in the basal and stress-induced vasopressin mRNA value in the parvocellular, but not the adjacent magnocellular division of PVN, is observed in adrenalectomized rats, whereas it returns to the basal level after glucocorticoid administration (Kovacs et al. 2000). In the human, it is assumed that hyponatremia followed by adrenal insufficiency is mainly due to the excessive production of vasopressin. In hypothalamic organotypic slice culture, it has been confirmed that dexamethasone treatment significantly suppresses the vasopressin gene mRNA in PVN, but not SCN (Kuwahara et al. 2003.) This *ex vivo* study also indicates that the suppressive effect on the cAMP-induced transcription activity by dexamethasone occurs in the vasopressinergic neurons themselves, independently of the synaptic interaction with the projected neurons.

It is ordinarily expected that the lack of CRH results in blunted or absent ACTH secretion. Interestingly, no significant difference of plasma ACTH concentration is observed under the basal condition between CRH-knockout and wild-type mice (Muglia et al. 1995). The mRNA expression of proopiomelanocortin (POMC), which is a large precursor of ACTH, is not impaired in corticotroph of CRH KO mice. Pituitary histology of knockout mice appears normal. On the other hand, the decrease of pituitary ACTH contents is approximately 70% of those of wild-type, probably reflecting the slight depletion of ACTH stores in corticotroph (Muglia et al. 2000). These observation suggest that CRH is not necessarily required for the basal expression of ACTH. Furthermore, vasopressin does not contribute to maintain the POMC gene expression in CRH deficiency, because the increase of the vasopressin immunoreactivity is unrecognized in PVN (Muglia et al. 1995). It is of interest that the gel filtration analysis shows that the unprocessed large precursor of ACTH accounts for a high percentage of immunoreactive ACTH in the anterior pituitary in CRH KO mice, probably because CRH serves as an activator of processing enzymes such as prohormone convertase 1/3 (PC1/3) which produces the authentic-sized, bioactive ACTH (Muglia et al. 2000; Fukuda et al. 2004). Unlike basal ACTH secretion, the stimulatory effect of plasma ACTH in CRH KO is smaller than that in wild-type, after pain stress. This result indicates that CRH plays an indispensable role in the induction of the ACTH release, especially under the stress condition.

The plasma corticosterone level in CRH-deficient mice is significantly lower in comparison to wild-type animals (Muglia et al. 1995). Moreover, severe atrophy of the zona fasciculata of the adrenal cortex where corticosterone is produced is observed in male CRH knockout mice. In female knockout mice, the extent of this hypoplasia is less than that of male knockout mice. On the other hand, the zona glomerulosa, where mineralocorticoid is mainly produced, and the adrenal medulla are histologically normal. After restraint stress followed by ether exposure, lower plasma corticosterone level of CRH-deficient mice is observed, compared with the wild-type. In particular, male knockout mice exhibit an extremely impaired ability to secrete corticosterone. The plasma corticosterone remains at the minimal level at the circadian nadir in normal rodents. This gender difference of adrenal responsiveness may be caused by the effect of sex hormones on the HPA axis. In addition, despite hypoglycemia by food deprivation, corticosterone concentration is not elevated in knockout mice. Altogether, the stress response is markedly reduced in CRH KO mice. In addition to the atrophy of the zona fasciculata, it is possible that the increase of unprocessed ACTH precursor affects the corticosterone production to some extent.

From a different viewpoint, it is an unexpected observation that basal ACTH is not elevated at all in male CRH KO mice despite adrenal insufficiency, because their corticosterone level is low. Likewise, no elevation of basal circulating ACTH is observed in the adrenalectomized CRH-deficient mice. Notably, exogenous CRH injection rapidly induces the ACTH secretion in these adrenalectomized mice, suggesting that CRH is essential to the ACTH secretion rather than the POMC gene transcription. Alternatively, the expression of CRHR1 in corticotroph may be significantly up-regulated in the CRH deficiency.

The expression of the vasopressin and CRH gene in the PVN parvocellular neurons is induced by the pro-inflammatory cytokines. There are several reports regarding the interaction between CRH/vasopressin and cytokines by the directly peripheral or cerebral administration of cytokines *in vivo*, or the *ex vivo* hypothalamic culture system. It is well-known that an excessive production of vasopressin induces the “syndrome of inappropriate secretion of antidiuretic hormone (SIADH),” which is characterized by hyponatoremia with excessive sodium excretion from the kidney (Bartter and Schwartz, 1967; Robertson, 2006; Ellison and Berl, 2007). Indeed, interleukin-6 (IL-6) leads to the excessive production of vasopressin in the hypothalamus in patients with meningitis or encephalitis, often causing SIADH (Baylis, 2003). In the dissociated fetal hypothalamic cell culture derived from the Fisher (F344/N) rat, 24h exposure of IL-6 stimulates vasopressin release (Wei et al. 2002). In this paper, IL-1β, TNFα, and lipopolysaccharide also increase the vasopressin secretion in a dose-dependent manner. In humans, plasma vasopressin levels are elevated 2h after IL-6 injection (Mastorakos et al. 1994). Interestingly, a recent analysis using DNA microarray shows a robust expression of IL-6 in rat SON after a 48h water deprivation (Ghorbel et al. 2003). The authors confirmed the coexistence of vasopressin and IL-6 in SON of the dehydrated rat, suggesting that IL-6 promotes the induction of the vasopressin expression and/or secretion through the autocrine/paracrine system. Consistent with the above findings, most of the vasopressin-positive cells in PVN and SON are co-localized with IL-6, whereas the expression pattern of the CRH-positive cells depends on the type of cells (Gonzalez-Hernandez et al. 2006).

It is also known that exposure to cytokines augments the expression and/or secretion of CRH. Infusions of IL-1 induced a significant secretion of hypothalamic CRH (Sapolsky et al. 1987; Berkenbosch et al. 1987). The intraperitoneal injection of IL-1α/β stimulates hypothalamic CRH mRNA expression and release (Suda et al. 1990). IL-1β increases CRH secretion from dispersed fetal rat hypothalamic cells, whereas this stimulatory effect is abolished by the treatment of protein kinase A (PKA) and protein kinase C (PKC) inhibitor (Hu et al. 1992a). Also, the endogenous CRH mRNA expression is induced by IL-1β along with the induction of c-*fos*, c-*jun*, and GR gene expression in human hepatoma NPLC-KC cells (Gill et al. 1998). On the other hand, IL-6 also increases the CRH gene expression in hypothalamic explants (Lyson and McCann, 1993), dissociated amygdalar cultures (Kasckow et al. 1997), and human endometrial cells (Makrigiannakis et al. 1999). IL-6, IL-1β and TNFα stimulate CRH secretion in dissociated hypothalamic culture (Wei et al. 2002). Thus, these observation indicate that the administration of IL-1α/β, IL-6 and other cytokines can promote the induction of the transcription and/or secretion of CRH/vasopressin *in vivo*. However, it is unclear whether these cytokines directly stimulate the PVN neurons, or indirectly stimulate them through the afferent neuronal inputs from other neurons, after perceiving the stimuli of these cytokines. Notably, the expression of cytokines in the brain is activated not only by inflammation, but also by a wide variety of stresses such as dehydration, restraint, swimming, or emotions. These cytokines may have a pleiotropic effect over the immune response. The peripheral administration of IL-1α/β induces plasma IL-6, suggesting that there exist complicated signaling cascades between the cytokines (Libert et al. 1990). Thus, brain cytokines have the ability to carry the various kinds of stress signals in the central nervous system, like hormones or neurotransmitters. For example, the following interesting observations regarding the relationship between IL-6 and the HPA axis using CRH-deficient mice have been reported (Bethin et al. 2000). After IL-6 injection, plasma ACTH increases in wild-type mice; however, a greater increase of ACTH is observed in CRH KO mice. The authors confirmed the expression of IL-6 receptor in the pituitary corticotroph and adrenal cortex. After lipopolysaccharide injection, the plasma corticosterone level is lower in either CRH or IL-6 knockout mice than in the wild-type; however, this stimulatory effect is almost unrecognized in CRH and IL-6 double knockout mice. These serial results indicate that IL-6 up-regulates the activation of the HPA axis alternative to CRH. Thus, IL-6 activates the HPA axis through a CRH-dependent pathway and an independent pathway. All together, the profound relationship between vasopressin/CRH and cytokines develops the immune-endocrine cross-talk system, supporting not only the immune response, but also wide-ranged, life-saving reactions to life-threatening environmental changes.

## Molecular Mechanisms of Vasopressin and CRH Gene Transcription

**Figure 1:**
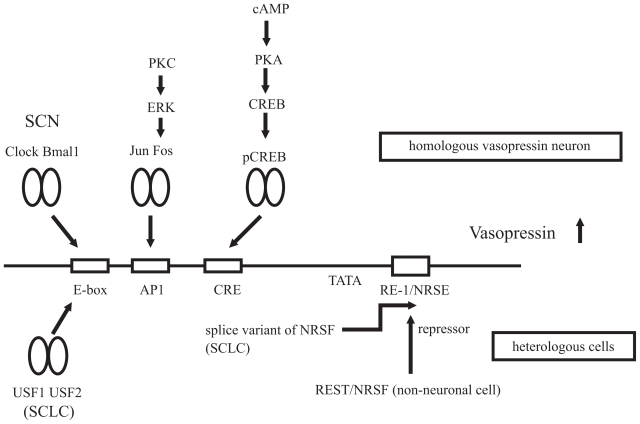
Proposed model of the molecular mechanisms of the vasopressin gene transcription A variety of transcription factors and signaling pathways are involved in the transcriptional regulation of the vasopressin gene. In the homologous vasopressinergic neurons in the hypothalamus, cAMP acts as an important second messenger of the vasopressin gene activation. After receiving the stresses, the activation of the cAMP/PKA pathways promotes the phosphorylation of CREB. This phosphorylated CREB activates the vasopressin promoter activity through the CRE site. The immediate early genes, Fos/Jun family proteins, also induce the vasopressin gene transcription by direct binding to the AP1 element, in response to various kinds of stimuli. In SCN, the Clock and Bmal1 heterodimer generates the circadian regulation of the vasopressin gene via the E-box element. On the other hand, REST/NRSF represses the vasopressin promoter activity via the RE-1/NRSE site in the heterologous cells. In contrast, in some cancer cells, such as small cell lung carcinoma (SCLC), the splice variant of REST/NRSF and/or USF1/USF2 induces the ectopic expression of the vasopressin gene.

**Figure 2:**
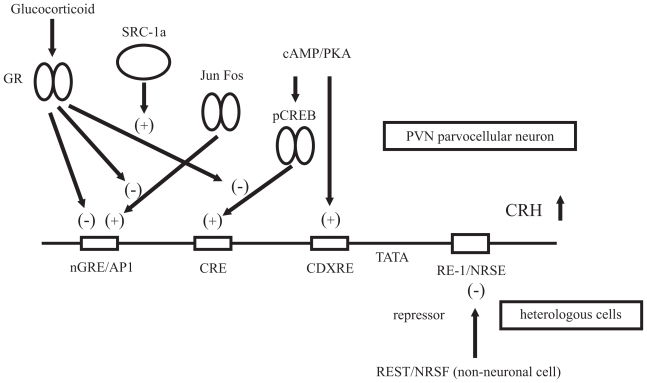
Proposed model of the molecular mechanisms of the CRH gene transcription A variety of transcription factors and signaling pathways are involved in the transcriptional regulation of the CRH gene. The cAMP/PKA signaling cascade acts as an important second messenger of the CRH gene activation. The promoter activity of the CRH gene is induced by the phosphorylated CREB via the CRE site. The CDXRE region is also thought to be a responsible site. It is possible that the Fos/Jun family proteins activate the CRH gene transcription in response to various kinds of stimuli via the putative AP1 site. In addition, in PVN, CRH gene promoter activity is negatively regulated by GR, which directly binds to nGRE and/or interacts with another nuclear factor on CRE. In the latter case, SRC-1a is a candidate protein which supports this suppressive effect of GR. On the other hand, in the heterologous cells, REST/NRSF inhibits the random expression of the CRH gene as a silencing factor.

The vasopressin and CRH gene expression is activated by numerous physiological stimuli, as described above. Because the roles of these hormones are closely linked to life homeostasis, it becomes necessary to establish an accurate and stringent gene regulation system. For example, clinically, excessive production of vasopressin is known to cause SIADH, whereas excessive CRH causes Cushing disease, depression, and anorexia nervosa. To understand the gene expression of both neuropeptides, numerous detailed promoter analyses have been carried out. Several important *cis*-elements in the 5′-promoter region and intracellular signaling cascades have been identified (Fig. 1 and 2).

### cAMP/PKA

It is generally accepted that cAMP is an important positive inducer of the vasopressin gene transcription. There is much evidence that the cAMP/PKA activation leads to the immediate induction of the vasopressin gene expression. The marked elevation of the intracellular cAMP is observed in SON during a hyperosmotic stimulation (Carter and Murphy, 1989). The phospholylation of CRE-binding protein (CREB) is rapidly induced in SON after osmotic stimulus (Shiromani et al. 1995). In addition, the cAMP activator elevates the vasopressin gene expression in fetal hypothalamic culture (Hu et al. 1993) and dissociated hypothalamic primary culture (Wei et al. 2002). Precise reporter analyses using the heterologous cell lines show that cAMP stimulator also augments vasopressin gene transcription (Pardy et al. 1992; Emanuel et al. 1992). Two CREs have been identified in the rat vasopressin 5′-promoter region (−227/−220 and −123/−116) using placenta-derived JEG3 cells (Iwasaki et al. 1997). In this paper, co-transfection with the PKA catalytic subunit (a constitutive active form of PKA) or CREB expression vector significantly induces the vasopressin gene promoter activity, as well as cAMP stimulators. However, the direct binding of CREB proteins to these putative CREs has not been verified by electrophoretic mobility shift assay (EMSA) or chromatin immunoprecipitation (ChIP).

It is known that the activation of CREB is mediated by Ca-dependent pathway, as well as a cAMP/PKA signaling pathway. Calcium/calmodulin-dependent protein kinase (CaMK) II activates the CREB proteins through phosphorylation at the portion of Ser133 (Sheng et al. 1991; Dash et al. 1991). However, to date, there is no report that the Ca influx induces the vasopressin gene promoter activity.

The CRH gene promoter activity is also enhanced through the cAMP/PKA-dependent pathway. The CRH gene expression and release are activated by the cAMP/PKA-dependent pathway using hypothalamic explants (Suda et al. 1990). The activation of the PKA signaling also increases the CRH gene expression in rat fetal hypothalamic cultures (Emanuel et al. 1992), or primary amygdalar cultures (Kasckow et al. 1997). Moreover, cAMP activator stimulates the endogenous CRH mRNA expression and secretes CRH in culture medium in homologous BE(2)M17 (Kasckow et al. 1994) and BE(2)C cell lines (Kasckow et al. 1995). The gene expression of endogenous CRH is also up-regulated in the immortalized hypothalamic (Nikodemova et al. 2003) and amygdalar (Mulchahey et al. 1999) cell lines.

An important CRE site has been identified in the human CRH promoter region (−228/−221), using neuroblastoma SK-N-MC and choriocarcinoma JAR cell lines (Spengler et al. 1992). The cAMP-induced CRH promoter activity is reduced in the deletion construct which does not contain CRE. When only single or multiple copies of this CRE (−228/−221) were fused to tk promoter, forskolin treatment also induced the transcription activity of these heterologous promoters. The sequence of CRE in the CRH promoter, TGACGTCA, is completely matched to the consensus CRE. In addition, a novel cAMP-responsive element, caudal-type homeobox response element (CDXRE), has also been found in the proximal promoter region of CRH (−125/−118) (King et al. 2002). According to this paper, the cAMP-stimulating effect of the CRH promoter activity is not abolished in the mutated construct in which only CRE is disrupted in AtT20 cells. It is possible that both CRE and CDXRE participate in the cAMP-mediated CRH gene promoter activity.

### AP1/PKC

In contrast to the cAMP-mediated pathway, the role of the Fos/Jun family proteins in the vasopressin gene regulation is still obscure. Recently, our group identified a functional activation protein 1 (AP1) element (−134/−128, TGAATCA) in the rat vasopressin promoter using human neuroblastoma BE(2)M17 cells (Yoshida et al. 2006). The AP1 protein consists of five Fos proteins (c-Fos, Fra-1, Fra-2, FosB, and its natural truncated form ΔFosB), and three Jun proteins (c-Jun, JunB, and JunD) (Herdegen and Waetzig, 2001). The Fos/Jun family of proteins is known as an immediate early gene in neuronal cells, because the expression is induced rapidly in response to diverse extracellular stimuli. For example, c-Fos is widely used as a stress marker in neurons. These proteins bind to a specific sequence (TGAC/GTCA; known as AP1) of the promoter region of the target genes, by forming a homodimer (Jun/Jun) or heterodimer (Fos/Jun). Indeed, the Fos/Jun family proteins mediate the stress-induced transcription activity of many genes in the endocrine system, including ACTH (Boutillier et al. 1995), prolactin (Caccavelli et al. 1998), and follicle-stimulating hormone (FSH) (Strahl et al. 1997).

Many previous studies have shown that the Fos/Jun family proteins are rapidly expressed in the vasopressinergic neurons in response to a variety of extracellular stimuli, synchronizing with the activation of vasopressin gene expression. According to the type of stress, such as hypertonic saline (Sharp et al. 1991; Shiromani et al. 1995; Carter and Murphy, 1990), lipopolysaccharide (Rivest and Laflamme, 1995; Xu et al. 2003), or photic stimulation (Francois-Bellan et al. 1999), differential expression patterns (both spatial and temporal) of Fos/Jun family proteins are observed. In the vasopressinergic neurons in the SON and PVN, the acute osmotic stimuli increase c-*fos*, c-*jun*, and *junB* mRNAs (Luckman et al. 1996). Sustained expression of Fos family proteins was also reported during chronic hypertonic saline treatment (Miyata et al. 2001). These results strongly suggest that the Fos/Jun family proteins mediate the rapid transcriptional induction of the vasopressin gene by extracellular stimuli. The AP1 site we identified is the sole responsible element for Fos/Jun proteins, because the AP1-induced potent transcription activity of the vasopressin gene is completely abolished when the AP1 site is altered. The expression pattern of each Fos or Jun protein is different, depending on the type of stress. The homodimers or heterodimers of Fos/Jun proteins bind to the AP1 site with different affinities (Nakabeppu et al. 1988). Therefore, various kinds of stress may lead to the generation of the divergent and complicated regulation pattern of vasopressin gene expression via the AP1 proteins.

The involvement of the protein kinase C (PKC) pathway in the vasopressin gene promoter activity still remains unclear (Hu et al. 1992b; Hu et al. 1993; Wayte et al. 1997; Wei et al. 2002; Arima et al. 2003). However, because the activation of the Fos/Jun family proteins is deeply associated with the PKC pathway, we hypothesized that the activation of PKC increases the vasopressin gene promoter activity through the c-Fos-mediated pathway. Our group showed the possibility of this hypothesis using BE(2)M17 cells (Yoshida et al. 2006). A PKC activator, 12-O-tetradecanoyl phorbor 13-acetate (TPA), increased the vasopressin gene promoter activity. This stimulatory effect is significantly decreased by the elimination of the AP1 site, or blocked by the treatment of the specific extracellular signal-regulated kinase (ERK) inhibitor, or PKC inhibitor. The promoter region of the *c*-*fos* gene contains the serum response element (SRE), the *sis*-inducible element (SIE), and CRE (Treisman, 1995). The *c-fos* gene expression can be induced rapidly by a wide variety of extracellular stimuli through these *cis*-regulatory elements (Jacknecht, 1995). In particular, the SRE element is an important region of the PKC-induced transcription of the *c*-*fos* gene, mediated by the activation of the ERK signaling cascade. Our result implies that the vasopressin gene promoter activity is enhanced through the activation of the PKC-ERK-c-Fos signaling pathway.

The role of the PKC pathway or AP1 proteins in CRH gene regulation is still controversial. TPA stimulates the CRH gene expression in heterologous NPLC cells through the elongation of the additional poly (A) tail (Adler et al. 1992). TPA, as well as Fsk increases the CRH secretion from hepatoma-derived, homologous NPLC-KC cells (Parkes et al. 1993), or from dissociated hypothalamic primary culture (Wei et al. 2002). Other studies have shown that microinjected TPA does not significantly enhance the CRH mRNA value (Itoi et al. 1996). As with vasopressin, the Fos/Jun family proteins are rapidly expressed in the CRH neurons in response to a variety of extracellular stimuli, suggesting that AP1 proteins activate the CRH gene expression. The co-expression of *c*-*fos* or c-*jun* increases the promoter activity of CRH gene via two putative AP1 elements in the 5′-flanking region of the CRH gene (Malkoski and Dorin, 1999) (see below). On the other hand, pre-treatment of *c-fos* antisense oligonucleotide fails to suppress the hypoglycemia-induced CRH gene expression in the hypothalamus (Itoi et al. 1996).

### Glucocorticoid

GR up-regulates the transcription activity of many target genes at the glucocorticoid response element (GRE), consensus GGTACANNNTGTTCT (Lieberman, 2007). Many investigators have made efforts to clarify the molecular mechanism of the negative regulation by glucocorticoid in the vasopressin or CRH gene transcription. To explain the negative feedback system, two hypotheses have been advocated: 1) GR dimer suppresses the promoter activity by directly binding to the so-called negative GRE (nGRE). 2) The interaction between GR and other transcriptional factors or cofactors inhibits the binding of these nuclear factors to the specific *cis*-acting element. In fact, the promoter sequences of many genes whose expression is negatively regulated by glucocorticoid, does not always contain GRE or nGRE. In the majority of these genes, GR can attenuate the promoter activity by a squelching or tethering effect to other factors without binding directly to GRE. Putative GRE was pointed out in −622/−608 of the rat vasopressin promoter region (Mohr and Richter, 1990). However, there have been various opinions regarding the suppressive element by glucocorticoid in the vasopressin gene. Putative GRE is located within the region −300 to −155 in the bovine vasopressin promoter sequence by EMSA (Burke et al. 1997). On the other hand, in the deletion analysis using placenta-derived JEG3 cells, the responsive element for the suppressive effect by dexamethasone on the cAMP-induced promoter activity is present in −102 to −36 of the rat vasopressin promoter region (Iwasaki et al. 1997). This suppressive effect still remains in the mutated construct in which two putative CREs are eliminated, suggesting that glucocorticoid reduces the vasopressin promoter activity independently of CREs. Otherwise, putative GRE is −628 in the vasopressin gene promoter region (Kim et al. 2001). The vasopressin gene promoter activity is suppressed through this element, after dexamethasone treatment.

Only the 18bp CRE-containing region (−232/−215) serves as a responsive site for the suppressive effect by glucocorticoid on the cAMP-induced human CRH gene promoter activity in corticotroph AtT20 cells (Guardiola-Diaz et al. 1996). On the contrary, functional negative (n) GRE sites are identified in the 5′-flanking region (−278/−249) of the CRH gene (Malkoski and Dorin, 1999). Three serial GRE half-sites are recognized in the above short sequences, all of which are involved in the negative regulation of the CRH gene transcription by the direct binding of GR. 8-bromo cAMP-induced CRH gene promoter activity is significantly reduced by glucocorticoid in AtT20 cells; however, this suppressive effect is abolished in the deleted or mutated construct lacking these nGRE site(s). According to this paper, two AP1 sites are located adjacent to the GRE half-sites. The c-Fos- or c-Jun-induced promoter activity of the CRH gene is significantly repressed by dexamethasone treatment, probably due to the close proximity of AP1-like sites and GREs (partly overlapping). On the other hand, this repressive effect is exerted by the interaction between nGRE and CRE (King et al. 2002). As expected, this effect is abolished in the deletion construct which contains CRE, but not nGRE, whereas this effect is also lost in the mutated construct in which only CRE (−228/−221) is altered. In addition, glucocorticoid stimulates the transcription activity in the minimal homologous or heterologous promoter, which is constructed by the insertion of the isolated CRE. These observation suggests that CRE serves as a main responsive site for the inhibitory effect by glucocorticoid in the presence of nGRE. EMSA analysis shows that CREB and c-Fos can bind CRE in AtT20 cells, and that the binding ability of these nuclear factors is reduced by the treatment of dexamethasone together with cAMP, implying that negative regulation of the CRH gene is caused by the protein-protein interaction between CREB, c-Fos, or other unknown factors and GR on the CRE site. There is another paper dealing with the importance of CRE in the negative regulation by glucocorticoid (Yamamori et al. 2007). The forskolin-stimulated CRH gene promoter activity is suppressed by dexamethasone in the BE(2)C cells which endogenously express CRH. This inhibitory effect is ablated by the elimination of CRE, but not nGREs.

There is an interesting report about the interaction of GR and a coactivator in the CRH gene regulation by glucocorticoid (van der Laan et al. in press). According to this latest paper, when steroid receptor coactivator 1a (SRC-1a) is over-expressed in AtT20 cells, the suppressive effect by dexamethasone on the Fsk-induced CRH gene promoter activity is enhanced, compared with the untransfected cells. Conversely, dexamethasone increases the Fsk-induced CRH gene promoter activity, when the SRC-1a isoform, SRC-1e, is introduced. The SRC family members are highly expressed in the brain and have a distinct expression pattern: SRC-1a mRNA is strongly expressed in the magnocellular and parvocellular parts of PVN and SON, whereas the expression level of SRC-1e mRNA is very high in the amygdala and much lower in PVN (Maijer et al. 2000). These observation suggest that the negative regulation of the CRH gene expression in PVN is mediated by SRC-1a. On the contrary, as mentioned above, the stimulating (or at least, nonsuppressible) effect on the CRH gene expression by glucocorticoid in the amygdala is probably due to the different expression pattern of SRCs. SRCs are known to act as a cofactor in cooperation with a variety of nuclear receptors or transcription factors (Xu and Li, 2003), such as AP1 (Lee et al. 1998) or nuclear factor kB (NFkB)(Na et al. 1998). Our group has also shown the preliminary data on the enhancing effect by SRC-1a on the vasopressin gene transcription via AP1 site (Yoshida et al. 2006).

GR interacts with numerous transcription factors and intracellular signaling cascades, such as AP1, CREB, NFkB, NGFI-B/Nurr77, STAT family members, and MAPKs, achieving the multipotential effect of glucocorticoid (Liberman et al. 2007). It is possible that GC reduces the CRH or vasopressin gene promoter activity, due to the inhibition of the activation of these transcription factors or signaling pathways at the transcriptional or posttranscriptional level. Otherwise, the acute non-genomic effect of glucocorticoid may support the rapid suppression of this hypothalamic hormone gene expression.

### Circadian regulation

Vasopressin-positive fibers are observed in SCN, which is known to be a circadian generator. In SCN, the transcription of the vasopressin gene receives a unique circadian regulation by photic stimulation (Jin et al. 1999). This intrinsic circadian oscillation of the vasopressin gene expression is still preserved in removed, organotypic slice cultures (Arima et al. 2002). The physiological significance of vasopressin in this region is unclear; however, it is possible that vasopressin in SCN influences the circadian rhythm of other brain peptides via synaptic interaction, as a pacemaker. In SCN, the heterodimer of Clock and Bmal1 generates the circadian regulation of the vasopressin gene transcription via the CACGTG E-box element (Jin et al. 1999).

On the other hand, the CRH gene expression is undetectable in SCN. In contrast, the circadian regulation of the CRH mRNA expression is recognized in the mammalian PVN (Kwak et al. 1992; Kwak et al. 1993). Therefore, the circadian variation of POMC gene expression in pituitary corticotroph is assumed to be derived from the circadian variation of CRH in PVN. This cyclic expression of CRH is not abolished in adrenalectomized rats and it is preserved after the constitutive replacement by the corticosterone pellet, indicating that circulating glucocorticoid is not involved in the circadian regulation of CRH (Watts et al. 2004). In addition, the circadian rhythm of plasma ACTH and corticosterone concentration becomes lost in male and female CRH KO mice (Muglia et al. 1997). However, constitutive replacement of CRH (constant infusion by osmotic minipump) repairs the circadian variation of the HPA axis. This result suggests that the presence of CRH is required for the formation of the diurnal regulation of the HPA axis, but the circadian variation of CRH is not indispensable, at least in rodents. The circadian variation of the HPA axis may be generated by the cooperation of CRH and another circadian modulator, for example, the neuronal inputs from SCN.

### USF1/USF2

The vasopressin gene expression is limited to particular regions, even in the brain area. In contrast, in some cancer cells, such as small cell lung carcinoma (SCLC), the constitutive vasopressin gene expression is recognized and often causes SIADH. Unfortunately, the determinant of the tissue-specific expression of the vasopressin gene has not been fully elucidated. Upstream stimulatory factor 1 (USF1) and USF2 contribute to the ectopic vasopressin gene expression in the SCLC cells, by the binding of the E-box element in the vasopressin gene promoter region (Coulson et al. 1999a). USF1/USF2, which is a basic-helix-loop-helix (bHLH) transcription factor, is ubiquitously present in a broad range of cells (Sirito et al. 1994). It preferentially binds to the E-box site and induces the promoter activity of target genes.

### REST/NRSF

USF1/USF2 is not a determinant which facilitates the vasopressin gene expression in the SCLC cells in particular, and not other non-neuronal cells, because these transcription factors are ubiquitously expressed. To clarify the mechanism of the tissue-specific gene expression, much more information about the epigenetic gene regulation system or silencing factors such as RNA interference is probably required. Epigenetic regulation is defined as the heritable change of gene expression without alteration in DNA sequence, including chromatin modification, such as DNA methylation or histone modification. Thus, the epigenetic modification serves as the first main switch of gene expression, and resultantly, various transcription factors serve as an activator or inactivator. For example, the methylation pattern of CpG sites in the genome is known to be a key determinant of the gene expression. It is known that the aberrant methylation of the CpG island of oncogenes is deeply involved in tumorigenesis. However, the understanding of epigenetics is also important to explain the mechanism of tissue-specific expression of many hormones.

Unfortunately, we have no information regarding the differences in methylation patterns of vasopressin and CRH gene between homologous and heterologous cells. However, recently, it is being assumed that an interesting silencing mechanism exists in both genes in non-neuronal cells. In the non-neuronal cells, repressor element 1 (RE-1) silencing transcription factor/neuron-restrictive silencer factor (REST/NRSF) represses the vasopressin gene expression by the direct binding of a neuron-restrictive silencer element (NRSE) (Ballas et al. 2005). However, the splice variant of REST/NRSF, produced in several kinds of SCLC cells, interferes with the normal silencing function of wild-type REST/NRSF, facilitating the ectopic expression of the vasopressin gene in the heterologous cells (Coulson et al. 1999b). According to recent findings, NRSF/REST represses the expression of many neuron-specific genes in non-neuronal cells, interacting with histone deacetylases, mSin3, and CoR-EST (Ballas and Mandel, 2005). The consensus NRSE motif (TTCAGCACCACGGAGCGCC) contains CpG dinucleotides which receive methylation in the differentiated non-neuronal cells, whereas the CpG island is poorly methylated in embryonic stem (ES) or neuron progenitor cells. It is possible that the differences in the methylation profiles of CpG sites in NRSE and its adjacent sequence are responsible for the gene silencing of the neuronal genes. In vasopressin gene expression, the epigenetic modification may also play an important role in the restricted expression.

The putative RE-1/NRSE site was previously predicted in the first intron of the CRH gene (Schoenherr et al. 1996). The sequence of this element is highly conserved (90%–95%) among all species, in contrast to the lower similarity of CRH gene intron except RE-1/NRSE. When RE-1/NRSE is disrupted, the basal promoter activity of the CRH gene is robustly elevated in myoblast-derived L6 cells in which REST/NRSF is abundantly expressed, compared with the wild-type construct (Seth and Majzoub, 2001). On the other hand, in neuronal PC12 cells, the promoter activity of the CRH gene is down-regulated by the overexpression of REST/NRSF. The inhibitory effect by REST/NRSF is lost when RE-1/NRSE is mutated. Interestingly, the opposite observation is obtained in another neuronal NG108 cell line. Thus, the promoter activity is stimulated in this mutant when REST/NRSF expression vector is introduced, indicating that REST/NRSF acts as an enhancer through the RE-1/NRSF-independent pathway, depending on the types of cells or tissues.

## Perspective

As discussed above, vasopressin and CRH play a crucial role in the appropriate response to environmental stimuli. Vasopressin and CRH possess diverse physiological functions, such as the maintenance of water balance and plasma osmolality, regulation of blood pressure, immune responses, energy homeostasis, feeding behavior, sexual behavior, emotion, and memory. They are thought to be fundamental and primitive reactions which have been conserved from comparatively lower organisms. These reactions can be divided into two groups: the static reaction such as the maintenance of equilibrium of the internal milieu, and the dynamic reaction such as active behavior to external milieu. All of these are indispensable for the survival and existence for all organisms. Both hypothalamic neuropeptides conduct these basic responses to facilitate the flexible adjustment to the environmental changes.

Because the physiological action of vasopressin and CRH are directly linked to the maintenance of homeostasis, their gene expression must be mediated stringently. The regulatory system is thought to be often highly conserved among species. Some important *cis*-elements have been discovered in the vasopressin and CRH gene promoter region. It is probably undeniable that the cAMP/PKA pathway activates the vasopressin and CRH gene transcription, because of the large amount of evidence attesting to it; however, the whole picture about other regulators has not yet been fully elucidated.

For example, there are still unsolved problems about the osmoregulatory system of the vasopressin gene expression. Indeed, many investigators reveal the possibility that several transcription factors or signaling pathways are involved in osmoregulation. However, after the osmoreceptor (or vasopressinergic neuron itself?) perceives the osmotic stimuli, would only the above candidate members generate the osmotic induction of the vasopressin gene? Proteomics analysis may provide us with a satisfactory solution to this problem. Recently, several up-regulated and down-regulated genes were identified in SON by chronic dehydration using DNA microarray (Gouraud et al. 2007; Qui et al. 2007). Among these gene profiling lists, novel factors may be discovered, ones which stimulate the synthesis or secretion of vasopressin. It is also possible that the involvement of mRNA stability may be added to the previous detailed promoter analyses. Several investigators have shown that osmotic stress induces the elongation of the poly (A) tail of vasopressin mRNA, resulting in the mRNA stability which promotes the vasopressin production (Carrazana, et al. 1988; Carter and Murphy, 1991).

There are different observations about the molecular mechanism of the negative feedback system by glucocorticoid, depending on the utilized cell lines. To date, no consensus about this problem has been obtained. Most of the promoter analyses have been performed using the mono-clonal culture cells, because the isolation of pure cells or neurons producing a particular hormone is extremely difficult. However, the observations made in tumor-derived cells are not always consistent with one another. For example, it may pose as an obstacle that tumor-derived cell lines utilized in the promoter analysis often lack the endogenous GR.

Moreover, the divergent vasopressin and CRH gene expression implies the participation of an unknown regulation system, such as a non-coding RNA or silencing factor. What role does the untranslated long intron play in the gene regulation system? Research regarding this novel gene regulation system has just begun. Tissue-specific expression of vasopressin and CRH may be under the epigenetic control. The methylation of the CpG island may possibly prevent the random ectopic expression of these genes. The osmosensitive factors may just be found among them. CRH was first discovered a quarter-century ago, and further, in the case of vasopressin, more than a half-century has already passed. Indeed, the progress in molecular biology has extended our knowledge regarding these hormones; however, there are still many difficult problems that need to be solved.
